# Identifying strategies to maximise recruitment and retention of practices and patients in a multicentre randomised controlled trial of an intervention to optimise secondary prevention for coronary heart disease in primary care

**DOI:** 10.1186/1471-2288-9-40

**Published:** 2009-06-19

**Authors:** Claire S Leathem, Margaret E Cupples, Mary C Byrne, Mary O'Malley, Ailish Houlihan, Andrew W Murphy, Susan M Smith

**Affiliations:** 1 Public Health Medicine & Primary Care, Queen's University Belfast, Belfast, Northern Ireland; 2 Department of General Practice, National University of Ireland, Galway, Ireland; 3 Public Health & Primary Care, Trinity College, Dublin, Ireland

## Abstract

**Background:**

Recruitment and retention of patients and healthcare providers in randomised controlled trials (RCTs) is important in order to determine the effectiveness of interventions. However, failure to achieve recruitment targets is common and reasons why a particular recruitment strategy works for one study and not another remain unclear. We sought to describe a strategy used in a multicentre RCT in primary care, to report researchers' and participants' experiences of its implementation and to inform future strategies to maximise recruitment and retention.

**Methods:**

In total 48 general practices and 903 patients were recruited from three different areas of Ireland to a RCT of an intervention designed to optimise secondary prevention of coronary heart disease. The recruitment process involved telephoning practices, posting information, visiting practices, identifying potential participants, posting invitations and obtaining consent. Retention involved patients attending reviews and responding to questionnaires and practices facilitating data collection.

**Results:**

We achieved high retention rates for practices (100%) and for patients (85%) over an 18-month intervention period. Pilot work, knowledge of the setting, awareness of change in staff and organisation amongst participant sites, rapid responses to queries and acknowledgement of practitioners' contributions were identified as being important. Minor variations in protocol and research support helped to meet varied, complex and changing individual needs of practitioners and patients and encouraged retention in the trial. A collaborative relationship between researcher and practice staff which required time to develop was perceived as vital for both recruitment and retention.

**Conclusion:**

Recruiting and retaining the numbers of practices and patients estimated as required to provide findings with adequate power contributes to increased confidence in the validity and generalisability of RCT results. A continuous dynamic process of monitoring progress within trials and tailoring strategies to particular circumstances, whilst not compromising trial protocols, should allow maximal recruitment and retention.

**Trial registration:**

ISRCTN24081411

## Background

### Introduction

Two of the most important challenges in clinical research are those of recruitment and retention [1-8]. Difficulty with recruitment can cause lengthy time delays to a research project. If not foreseen, this can cause problems with budgetary constraints, which may consequently lead to shortening the duration of the study intervention [9]. A recent report indicates that one third of trials are forced to seek additional funds due to recruitment delays [10].

As high proportions of patient contacts occur in primary care, e.g. 90% within the NHS [11], general practice offers the potential of access to large numbers of participants for research studies, but recruitment in this setting involves a particular succession of challenges. It is a two-step process, requiring firstly the consent of staff in an increasingly busy work environment where space and time are frequently limited, and then the consent and commitment of sufficient numbers of eligible patients. Previous reports suggest reasons why primary care practitioners and patients become involved in research. Practitioners' reasons range from deriving satisfaction in helping to establish correct treatment decisions to an opportunity for practice staff to participate in research: they may be attracted to participate in a study when they consider the research relevant and necessary, and the research study team experienced and supportive [12-15]. Patients' reasons include potential advantages in care received (for example longer consultation time and regular physical measurements), the attitude of researchers, the quality of information supplied and also altruism, hoping that their participation will benefit others [16,17].

It cannot, however, be assumed that all practitioners and patients will be interested in taking part in research. Some practitioners may have difficulty envisaging how their existing working practice could accommodate research activities [13] being concerned about the potential disruption to staff and patients, increasing demands on time [6,8,14,18], lack of support [19], the burden of rigorous data collection [20], adverse impact on the doctor-patient relationship, and reluctance to commit patients to incurring expenses in travel and time [19]. Patients' concerns include reconciling compliance with research protocols (e.g. follow up appointments) and demands of their lives, especially with caring responsibilities and work commitments, environmental factors (such as lack of transportation), unintelligible questionnaires and perceptions of unpleasant interventions: they may simply lack interest in research, have inadequate understanding of the study or mistrust the investigators. Patients who do participate in trials experience waning motivation over time [1,16,21,22].

Even when practices are committed to the study it is no guarantee that the recruitment and retention of study patients whose consensual participation is key to the success of the study will be straightforward [23]. Factors influencing recruitment also influence retention. Previous studies report little detail of how knowledge of barriers and facilitators to recruitment can be successfully translated into future strategies [8]. This paper aims to report the difficulties and successes experienced in attempting to apply previous knowledge to the recruitment and retention of participants in the 'SPHERE' study, a RCT of an intervention for secondary prevention in coronary heart disease [24] and to identify practical guidance for improving recruitment and retention of practitioners and patients to benefit future research studies [25].

### Setting and Sample

Coronary heart disease (CHD) remains one of the commonest causes of premature death worldwide and evidence suggests secondary prevention remains sub-optimal.

**Figure 1:**
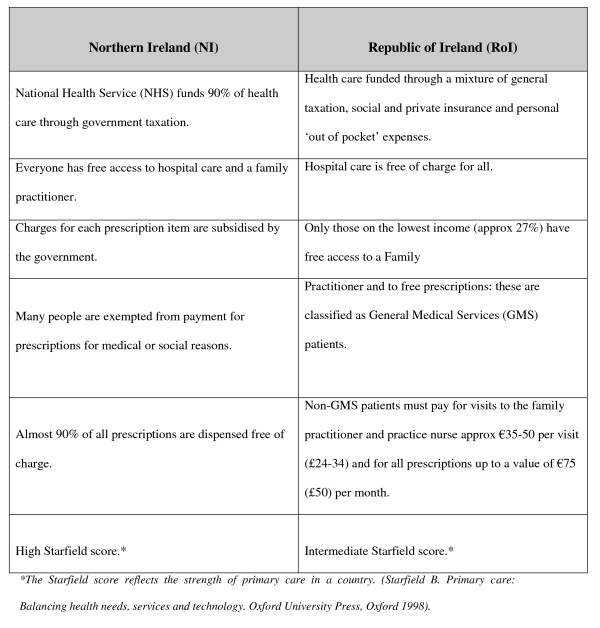
**Characteristics of Healthcare Systems in Northern Ireland and Republic of Ireland**.

The SPHERE study is a RCT of a tailored intervention to improve secondary prevention of CHD in general practice [24]. It is set in two study regions in the Republic of Ireland (RoI): West and East, and one in Northern Ireland (NI). The system of general practice organisation is different in RoI and NI (Figure 1) necessitating our recruitment strategy to be equally applicable to both healthcare systems. The study follows the MRC framework for developing and evaluating complex interventions [26]: this descriptive report highlights how preliminary findings inform a definitive trial and how details of the context of a trial are relevant to its evaluation.

During a pilot study of the intervention in four practices, two in RoI and two in NI, qualitative research [27] provided valuable insights into issues surrounding recruitment and retention. Such issues included the value of phone call contact for improving uptake, the shortage of space in premises, the need for strategies to deal with waning enthusiasm, clear protocol structures, patient non-attendance due to duplication in chronic disease management clinics and minimizing research workload for practitioners. These issues informed the design of our intervention and our approach to recruiting and retaining practices and patients. Following this pilot, the main RCT took place complemented by a parallel qualitative evaluation of the intervention with a purposive sample of patients (n = 67), practitioners (n = 26) and research nurses (n = 4) (Corrigan M, D'Eath M, Cupples ME, Wilson J, Murphy AW. *Participants' experiences of a complex intervention for the secondary prevention of heart disease in general practices in Ireland – the Sphere study*. Submitted). A small element of this qualitative arm explored perceptions of the recruitment process, data which helped to inform this paper. Research nurses also recorded their observations and participants' comments about the process during the trial.

## Methods

### Practice Recruitment

Practice recruitment began with the sourcing of a list of all potentially eligible practices from the local relevant health authority. Practices were eligible to take part if they had a practice nurse involved in general patient care, did not contribute to the pilot phase of the study, were not participating in 'Heartwatch' (a limited RoI government initiative for the secondary prevention of CHD) and had a minimum General Medical Services (GMS) list size of 700 (RoI) or National Health Service (NHS) list size of 1800 (NI). In NI the NHS offers free primary health care services to all and people register for care with a specific general practice; in the RoI free services are only provided to patients who are deemed to be 'GMS eligible', using criteria such as age and income [28]. Family practitioners in the RoI have formal lists of such patients but also treat others whose care is funded from other sources and do not have formal lists of these, hence the GMS list was used as an indicator of minimum practice size. Determining practice eligibility in the RoI involved the research nurses telephoning 205 practices from their lists to ascertain this information. In NI information about practices' NHS list sizes was available by contacting a central administrative office. Subsequently during initial invitation phonecalls, five practices on the list were found to be ineligible due to not employing a practice nurse but utilizing Health Trust treatment room nurses.

Practices identified within each region as being eligible for inclusion were assigned a number and a researcher independent of the SPHERE study listed them in random order using computer generated random numbers. The lists were also stratified in each region by the number of whole-time equivalent GP partners per practice.

### Initial Contact with Practices

Using the randomly ordered lists, potentially eligible practices were telephoned in sequence by the research nurses to achieve the target number needed for the study. The nurses avoided making these calls at especially busy times such as Monday mornings and Friday afternoons and asked reception staff for appropriate times to speak to a GP or practice manager, not wanting to use time slots reserved for patients. The purpose of the initial phone call was to confirm practice eligibility for participation in the study and ascertain their interest in receiving further information. Practitioners were given a brief explanation of the study and asked if they would like to receive the study information sheet. Mention was made of a minor financial payment (€1000/£700) in recognition of the additional costs in time and resources practices might incur, including making phone calls to ask patients to attend appointments, using a room for SPHERE consultations and collecting research data. Practitioners who expressed interest were posted a letter and the study information sheet, presented on one A4 sheet as opposed to multiple pages, for ease of reading. It included details of the projected practice workload and the extent of patient involvement whether randomly selected as an intervention practice or a control practice. Practices were contacted after ten days to ascertain decisions regarding participation. These decisions were recorded onto a database along with reasons for disinterest, if given.

The research nurse visited practices who expressed an interest in taking part in the study to explain the project more fully and provide the opportunity for practitioners to ask questions face to face. Visits were arranged at the practice's convenience, usually being held over lunchtime and an invitation was extended to all practice staff in recognition that the study would involve their cooperation and also to promote 'practice' (rather than individual staff) ownership of the study. Practice data (e.g. staffing information, computerisation and special interests) were collected onto a Practice Recruitment Form and the study eligibility criteria were confirmed. Specific needs or requirements that the practice had in relation to the study were also recorded for follow-up by the research nurse. A key member of staff was identified for further communication in order to clarify communication channels and avoid contacting other staff unnecessarily. Practices who wished to take part completed a form signed by each practitioner to indicate their agreement to participate. They then received a letter welcoming them to the study and were assigned a practice study number.

A small honorarium was given to reimburse practices once they had completed recruitment. This payment did not represent significant financial gain for practices but was intended as an acknowledgment or 'thank you' for their work. It was paid to the practice and not to individual practitioners who were central to carrying out SPHERE study consultations.

### Patient Recruitment

Patients eligible for inclusion in the study were those with a documented CHD diagnosis defined as: previous acute myocardial infarction confirmed by ECG, cardiac enzymes and/or serum troponin levels, angina confirmed by exercise stress test, angiogram or thallium scan, coronary artery bypass graft (CABG) or percutaneous transluminal coronary angioplasty (PTCA), without terminal illness or housebound. Potentially eligible patients were identified in NI by accessing the computer based CHD registers that already existed and were used in the NHS GP contract [29]. In the RoI the research nurses facilitated the recruited practices to generate lists of CHD patients. This involved searching both paper and computer records (involving five different computer software packages) for hospital letters and prescriptions, accessing other existing disease registers e.g. of diabetic patients, and consulting with practice staff about patients they could remember who had a cardiac event or procedure. Each practice was asked to recruit a minimum of twenty patients.

Eligible patients randomly selected from each practice's CHD register, using computer generated numbers from a remote location, were contacted by post. The mailing included a letter, signed by a named member of the practice staff, inviting participation in the study and the SPHERE study information sheet, with telephone numbers to contact the project manager or research nurse if wished. A questionnaire was also included containing a helpline number in case difficulties were encountered with completion, and a reply slip and stamped practice addressed envelope to indicate interest in participation (with a request for their telephone number if interested). For confidentiality reasons the SPHERE questionnaire contained the patient's study number (not name or address) to enable identification of returned questionnaires which were stored securely at the practice until collected by the research nurse. In order to keep an account of the patient recruitment process and questionnaire completion rate, details of the mailing were recorded on the Patient Progress Form; this form included all replies received and those who did not respond. Non-responders were followed up 10 days after mailing by a reminder letter and/or a phone call. Patients who responded positively were invited to attend an initial baseline consultation with their practitioner.

### Obtaining Consent and Collecting Baseline Data

The research nurse visited the practitioners prior to the first patient consultation to review the study protocol and discuss key study issues, including the process of obtaining patient informed consent, the purpose and format of quality assurance visits, and most importantly communicating the vital role the practitioner would play throughout the study in relation to interacting with patients and implementing the intervention. Practitioners received training on obtaining standardised clinical measurements including blood pressure, cholesterol, body mass index and waist hip ratios. A minimum of two baseline quality assurance visits with individual practitioners were undertaken by the research nurses during the initial and later patient consultations. Once the baseline measurements had been obtained for all twenty patients the practices were randomly divided into either control (usual care) or intervention allocations.

Based on findings of our pilot work, training on delivering the intervention (recalling participating patients and delivering a consultation at 4 monthly intervals for the duration of the study) was provided for intervention practices during two 90 minute in-house training sessions. The time and detail of this training was tailored to individual practice and practitioner needs.

### Retention strategy

The practicalities of recording and storing research data in the practices were addressed by providing a data collection form one page in length and a laminated colour coded reference list of CHD medications to aid practitioners' recording of patient's drugs within specified categories. A red storage box was offered to hold all study materials, with carry handles for easy transport and a 'handifile' to store paperwork. Other documentation provided included a SPHERE practice manual; this colourful convenient document detailed the study protocol and included detachable A4 laminated cards which set out pictorially each step of the consultations. The red storage box was designed following feedback from the pilot study where practice nurses identified that they did not always have a designated room for their clinics but were assigned any available room on a daily or weekly basis. All these measures helped to smooth the course of the study for busy practices, thus increasing the likelihood of retaining their participation.

Intervention practitioners received further quality assurance visits from the research nurses who observed and assessed randomly selected patient consultations in order to monitor and enhance the methodological quality measures of the health behaviour intervention, recording any deviations from the agreed standard [30]. The quality assurance visit form was also used to provide feedback to practitioners on the prescribed content within their consultations and comments were invited. Arrangements were made for follow-up contact from the research nurses who telephoned practices two weeks before patient visits were due both as a reminder and as an opportunity to discuss any problems or queries. The research nurses forwarded the QA forms to the study project manager who coordinated and supported their efforts and shared innovations experienced enhancing the smooth running of the study. The project manager produced a reader-friendly newsletter for distribution to intervention practices. This was designed to help reinforce the practices' position as an integral part of the entire study, provide information about the study progress and highlight relevant current issues in CHD.

While intervention practices by necessity received more support for retention than control practices, this was not viewed as a source of bias since practice support from the research team was an explicit component of the SPHERE intervention.

## Results

### Practice participation rates and reasons

**Figure 2:**
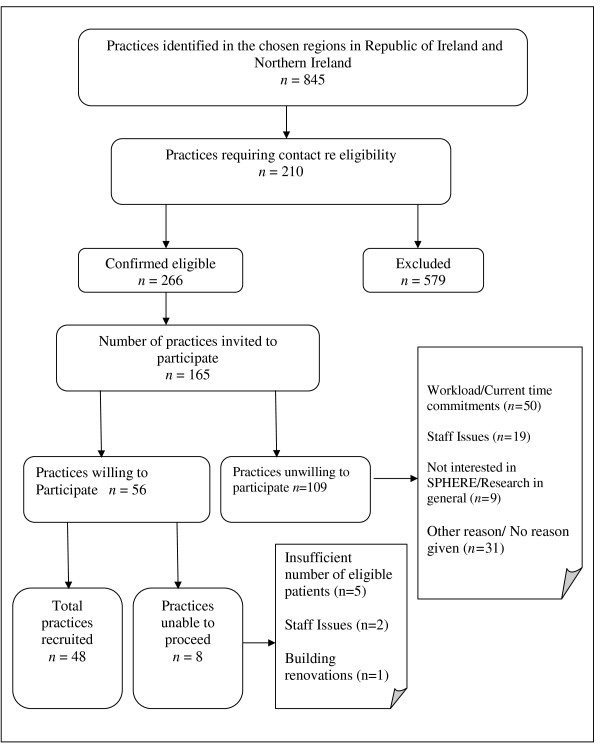
**Flowchart of Practices Approached and Recruited into SPHERE**.

**Figure 3:**
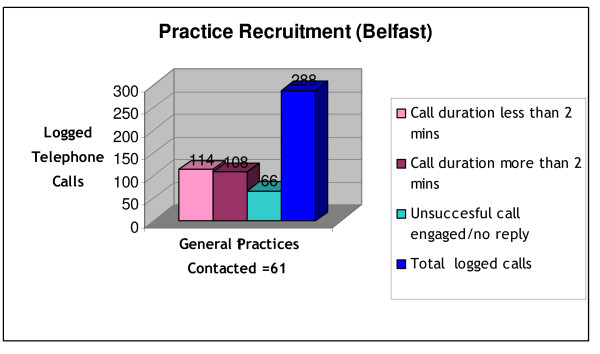
**Belfast Region Recruitment Phone Calls**.

A total of 845 practices were identified as potentially eligible, 711 in the RoI and 134 in NI. To recruit the target number of 48 practices (16 in each of the three regions), the research nurses invited, in sequence of random order, 165 of those which fulfilled the inclusion criteria: 109 declined, 56 who declared interest were visited and agreed to take part, but eight withdrew shortly afterwards for varying reasons (Figure 2), giving a participation rate of 33.9% (48/165). Recruitment of the target number of 48 practices took 12 months. Characteristics of participating practices are shown in Table 1. To recruit the 16 practices in NI a log record showed that a total of 288 phone calls were made, including unsuccessful attempts to speak to appropriate personnel (Figure 3). The reasons for practice non participation are shown in Figure 2.

**Table 1:** Characteristics of practices involved in SPHERE

	PRACTICES				
	
	Single Handed	Two Partner	Three Partner	Four Partner	> Four Partners
North	1	7	6	0	2

RoI East	8	3	1	4	0

RoI West	7	4	1	4	0

**Totals**	16	14	8	8	2

### Patient participation rates

We posted a total of 1795 invitation letters to patients and received 1492 (83%) responses (Table 2); 998 (55.6%) expressed an interest in participating and 903 (50.3%) attended baseline consultations and signed consent forms. Characteristics of non participating patients are shown in Table 3. The remaining 95 patients cancelled/did not attend appointments or were deemed ineligible; none of those who attended a baseline consultation declined study participation.

**Table 2:** Number of patients approached by practices

	PATIENTS				
	
	Invited	Declined	No Response	Agreed	Rate of recruitment
North	760	252	158	350	46.0%

RoI East	562	131	97	334	59.4%

RoI West	473	111	48	314	66.4%

**Totals**	1795	494	303	998	55.6%

**Table 3:** Patient non participant data

Variable	Participants (n = 903)	Non Participants (n = 772)	P	Difference [95% CI]
Male	69.9% (n = 631/903)	62.5% (n = 475/760)	0.001	7.4 [2.8, 11.9]
GMS (RoI only)	77.8% (n = 452/581)	81.7% (n = 313/383)	0.141	-3.9 [-8.9, 1.3]
Age [Mean (SD)]	67.480 (9.6) (total n = 903)	67.477 (10.7) (total n = 730)	0.995	0.003 [-0.998, 1.005]

### Retention issues

Throughout the study period practices experienced a variety of changes including key staff members changing jobs or retiring, seasonal increases in work loads such as "flu jab season" and the execution of necessary building work to premises. Patient attrition was caused by study visits to the practice becoming impracticable due to changes in circumstances or interest while others were unable to attend due to deterioration in their medical condition(s).

The crucial retention of both practices and patients was supported by the research nurses who attempted to minimise problems by sustaining practice contact both by telephone and practice visits to ensure ongoing needs assessments. They provided tailored support as appropriate, such as facilitating the practice through heavy work commitments, retraining new staff members, undertaking any extra administration duties generated by the study and helping contact and encourage patients who defaulted on appointments. Problems and solutions encountered were recorded electronically in a database in conjunction with quality assurance visits and monitored by the project manager. This ensured knowledge of treatment fidelity and facilitated practice support. Feedback was not only provided to practices through the Quality Assurance visits but also by the 'Practice Care Plan', which was designed to facilitate practitioners implementing the intervention and was updated as the study progressed. It included dates of QA visits and patient follow up consultations and recorded specific requests for support which the research nurse followed up. A copy was kept both at the practice and the research centre. Regular personal communication with the practices facilitated retention through the prompt resolution of their difficulties.

In qualitative interviews practitioners stated that the support received in dealing with queries in the early setting up stages and first patient consultations was very important. Regular phone calls, particularly the reminder calls were felt to be beneficial in helping practices to adhere to the study protocol and timeline. Knowing that they had good telephone access to the research nurses by direct contact numbers and having queries dealt with very quickly provided the necessary support they required. Practitioners valued the nurses being approachable, encouraging, helpful, friendly, reassuring and supportive. At study completion after at least 18 months, none of the 48 practices and 15% (135/903) of the patients who participated had withdrawn.

## Discussion

This paper reviews the experiences of recruitment and retention within a multicentre RCT delivered in primary care. It highlights the advantages of introductory telephone calls to practices, followed by postal information and face to face meetings in achieving high retention within a trial. We invested considerable effort in establishing and maintaining recruitment and retention, using resources which may not always be available to others, but the importance of identifying resources for these aspects of a research project should not be underestimated. Below we try to distil the key components of a successful strategy. The lessons to be learned may differ, depending on the healthcare setting in which future research is to be conducted.

### Initiating contact

The active recruitment measures used allowed personal contact between researchers and practitioners and avoided mailing unsolicited information, both of which are factors which encourage research participation [13]. The researcher's ability to engage the practitioner is central to positively facilitating decisions to participate and the vital ensuing relationship with the general practice team, especially when practices have not formerly participated in research. Previously reported practitioner recruitment rates [12] were higher (up to 91%) when personal meetings took place as opposed to recruitment by a telephone call alone (75%). However, 78% of practitioners recruited in that study were either friends or acquaintances of the study team, rather than randomly selected practitioners.

Asking healthcare providers to recruit patients following receipt of uninvited study information has been identified as a relatively unsuccessful method of recruitment [31]. Researchers who intend to recruit practices by posting an initial letter introducing the study are advised to ensure this is followed up with a timely phone call [13]. One RCT which asked physicians to deliver a smoking cessation intervention reported a participation rate of 9.8% when information was posted as opposed to 59% using face-to-face recruitment efforts [32].

### Providing information

The importance of an informed approach by the research team to recruitment of practices and patients cannot be underestimated [33]. Involving all practice staff in the visits allowed the research nurses to ensure that practices could be made fully aware of the not inconsiderable workload entailed in study participation for individual practice members and the commitment expected from patients. While this appeared to have a detrimental effect on our practice recruitment rate (33.9%) it proved a facilitator in the retention of both participating practices (100%) and patients (85%). Studies with high rates of patient recruitment cannot necessarily be perceived as superior to those with a lower rate [8] when other measures such as participant retention and treatment compliance also need to be taken into consideration. Practitioners who consent to participate in studies on the basis of inadequate information are more likely to withdraw early once the actual implications of participation become clear [15].

### Recognising time and place

The time spent by the research nurses at the outset visiting practitioners and providing training was thought to contribute to the fact that none of the patients who attended initial appointments at the practices declined participation. This, and the low rate of patient attrition, may be associated also with the initial posting of study information to patients, allowing them time to consider potential participation and discuss this with family or friends. Based on the pilot work findings, practices were encouraged to combine patient visits where possible if overlap with other chronic disease clinic attendance was identified, in order to avoid duplication of service provision and minimise patient expenses in travel and time.

It could be argued that patients with a past medical history of heart disease are sufficiently motivated to access medical interventions but previous research has reported that service uptake by these patients is less than optimal [34,35]. Also, using the practice address as a contact point for information and return of responses rather than an unfamiliar address was deemed to be more 'user friendly' for patients and kept the practice informed regarding replies received. Our findings suggested that this yielded a sample in which there was an absence of bias in response in respect of age and gender. We achieved a patient participation rate of 56%, considerably higher than the 38% response rate of patients with angina invited to participate in a recently reported trial [36].

### Involving practices

Our practice recruitment rate appeared lower than that (69%) in a previous study [3] which employed a similar process but recruited individual physicians, rather than practices. We required agreement by all partners within each practice, necessitating exclusion of some individuals who were willing to take part. Also, we required the practitioners to recruit the study patients and deliver the intervention whilst some studies supply their own clinical researchers to conduct patient enrolment and the study process does not increase the normal practice workload [37]. Our recruitment rate was similar to that reported previously in a cluster RCT exploring different methods of promoting secondary prevention of CHD (21 of 64 eligible practices; 32%) [38] but details of the support offered within that study were scant and it did not involve patient recruitment at the outset. The generalisability of study results may be improved if more practices with low levels of interest in research activity participate [9]. Direct financial recognition of individual practitioners' work associated with the research, rather than rewarding practices' participation in the study, may act as a more effective incentive. However, non-monetary incentives such as addressing practitioners' concerns and providing support through personal contact by the research staff may be equally encouraging [39].

### Challenges for the Future

Randomised trials continue to be an ever-increasing challenge in primary care due to difficulties in recruiting and retaining practices and patients. If the benefits of practice-based research are to be realised it is imperative that the challenges of adding research to a service that reports an expanding everyday workload [40] are minimised. Our experiences in the SPHERE study should inform future primary care-based research studies and help improve practice and patient participation and retention. We suggest consideration of the following key issues:

1 Designing a study with clinical relevance to primary care, in accordance with current service provision.

2 Carrying out a feasibility study to identify potential problems and create awareness of healthcare organisation in proposed research settings including pre-trial qualitative data to obtain opinions from patients, practitioners and ancillary staff in the context in which the trial is to be delivered.

3 Having the research team efforts coordinated by a designated Project Manager.

4 Providing information regarding projected workloads at the outset, especially if practitioners are required to recruit and consult with the study participants.

5 Assessing practice needs initially at the recruitment stage and ongoing throughout the study via a practice care plan.

6 Providing effective, sustained communication between the research team and practice staff and patients by phone calls prior to patient reviews and by extra visits when practices experience problems such as key staff members leaving.

7 Providing ongoing written information e.g. in the form of a study newsletter reporting on study progress, discussing issues which arise, and acknowledging the efforts of practice staff.

8 Assisting practices with administration generated by the study including helping contact defaulting patients and posting out appointments.

9 Recording study data by research nurse rather than practice staff where possible, avoiding potential for observation bias.

10 Working collaboratively and supporting practice staff, with speedy resolution of practice and patient queries.

11 Facilitating practitioners' and patients' study participation by ensuring all documentation provided is clear and user-friendly.

12 Financial acknowledgement of practice staff directly involved in the study.

## Conclusion

The potential benefits of primary care research to clinical outcomes are enormous. Facilitating the participation of a wide range of practices and patients in pragmatic research will allow increased confidence in the representativeness and generalisability of findings, with consequent positive impacts on patient care. The search for optimal methods of maximising practitioner and patient recruitment should continue.

## Competing interests

The authors declare that they have no competing interests.

## Authors' contributions

AWM, MEC and SMS acquired the funding and conceived the study with MCB participating in the design of the study intervention and data analysis. CSL participated in study intervention fieldwork and acquisition of data with AH & MOM and drafted the manuscript. All authors contributed to the critical revision of the manuscript.

## Pre-publication history

The pre-publication history for this paper can be accessed here:

http://www.biomedcentral.com/1471-2288/9/40/prepub
